# Benefits of hyperthermic intraperitoneal chemotherapy for patients with serosal invasion in gastric cancer: a meta-analysis of the randomized controlled trials

**DOI:** 10.1186/1471-2407-12-526

**Published:** 2012-11-16

**Authors:** Jingxu Sun, Yongxi Song, Zhenning Wang, Peng Gao, Xiaowan Chen, Yingying Xu, Jiwang Liang, Huimian Xu

**Affiliations:** 1 Department of Surgical Oncology and General Surgery, First Hospital of China Medical University, Shenyang 110001, China

**Keywords:** Hyperthermic intraperitoneal chemotherapy, Gastric cancer, Peritoneal recurrence, Meta-analysis

## Abstract

**Background:**

In this meta-analysis we aimed to determine the effectiveness and safety of hyperthermic intraperitoneal chemotherapy (HIPC) for patients with advanced gastric cancer who underwent gastrectomy.

**Methods:**

In accordance with standard meta-analysis procedures, our study included patients who underwent resection for advanced gastric cancer and were randomly allocated to receive either hyperthermic intraperitoneal chemotherapy or control. We searched PubMed (up to November 2011), EMBASE (up to November 2011), Cochrane Database of Systematic Reviews (CDSR), and Cochrane Central Register of Controlled Trials (CCTR) (up to November 2011). Both published and unpublished trials were included in the analysis, and no search restrictions were imposed. There was no language restriction. The results were analyzed using RevMan 5.1 software, which was provided by Cochrane Collaboration.

**Results:**

There were ten randomized controlled trials included in the analysis. A total of 1062 patients with gastric cancer in these studies were divided into the HIPC group (n = 518) and control group (n = 544). A significant improvement in survival was observed in the HIPC groups compared to the control group in the mitomycin C (MMC) subgroup (RR = 0.75, 95%CI 0.65-0.86; P < 0.00001) and the 5-FU group (RR = 0.69, 95%CI 0.52-0.90; P < 0.00001); the total RR was 0.73 (95%CI 0.64-0.83; P < 0.00001). Our findings indicated that HIPC potentially exhibited a lower peritoneal recurrence rate in the HIPC group compared to the control group (RR = 0.45, 95%CI 0.28-0.72; P = 0.001).

**Conclusions:**

Our meta-analysis demonstrated that HIPC may improve the overall survival rate for patients who receive resection for advance gastric cancer potentially, and help to prevent peritoneal local recurrence among patients with serosal invasion in gastric cancer.

## Background

Although significant advances have been achieved in recent years in experimental research, diagnosis, and treatment of cancer, gastric cancer (GC) remains the second most frequent cause of cancer death after lung cancer worldwide, and exhibits a poor prognosis [1,2]. Surgical resection plus extended lymph node dissection comprises the primary method of curative intent for localized gastric cancer, however, the 5-year survival rate remains unsatisfactory [3,4]. Peritoneal dissemination is one of the principal reasons for the recurrence and metastasis of gastric cancer in the peritoneal cavity, and it has been reported to be complicated and difficult to treat in recent years [5]. The peritoneal seeding of gastric cancer (GC) exhibits a high risk for patients who receive surgery alone, and systemic chemotherapy exhibits no significant effect [6]; the origins may be the free tumor cells from the primary gastric cancer that remain following surgery, or micrometastases in the peritoneal cavity [7].

In spite of the use of both systemic chemotherapy and radiation therapy, the survival rate of patients with advanced gastric cancer remains unsatisfactory. Adjuvant intraperitoneal chemotherapy (IPC) is recognized as an effective method to control peritoneal dissemination in GC patients who have undergone resection of the primary cancer [8,9]. Intraperitoneal chemotherapy is used to achieve longer survival by wiping out the mircometastases in the abdominal cavity and free tumor cells left after surgery that could not be cleaned up by intravenous chemotherapy. A number of studies have investigated whether intraperitoneal chemotherapy exhibits an effect on patients with advanced gastric cancer, such as Xu DZ et al. [10] and Yan TD et al. [8], and all reports reached a positive conclusion regarding improved survival rate. Recently, hyperthermia has been developed as an anticancer therapy, and has been demonstrated to exhibit a direct cytotoxic effect on tumor cells in the peritoneal cavity in conjunction with some anticancer chemotherapeutic agents [11]. Since Spratt et al. [12] reported the use of hyperthermic intraperitoneal chemotherapy for the treatment of peudomyxoma peritonei, several positive reports regarding hyperthermic intraperitoneal treatment for gastric cancer have been published, but the results were not unified. The purpose of our meta-analysis was to evaluate the effectiveness, safety, and preventive effects of hyperthermic intraperitoneal chemotherapy for patients with advance gastric cancer who received radical surgery through analysis of the results of randomized controlled trials.

## Methods

### Search strategy

An electronic search was applied to PubMed (up to November 2011), EMBASE (up to November 2011), Cochrane Database of Systematic Reviews (CDSR), and Cochrane Central Register of Controlled Trials (CCTR) (up to November 2011). Both published and unpublished trials were included, and no search restrictions were imposed. Furthermore, the reference lists of all selected studies were reviewed for further identification of potential relevant articles.

### Selection criteria

Inclusion criteria included all articles concerning patients with gastric cancer who were allocated randomly to receive surgery associated with intraperitoneal hyperthermic chemotherapy versus surgery without intraperitoneal hyperthermic chemotherapy. The advanced gastric cancer of the patients consisted of macroscopic serosal invasion without distant metastases or peritoneal carcinomatosis. Studies were limited to human trials, and there was no language restriction. If centers published duplicate trials with an increased number of patients or follow-up time period, we utilized the most complete reports in the meta-analysis.

### Date extraction and critical appraisal

Two reviewers (one clinical, Jingxu Sun, and one non-clinical, Xiaowan Chen) reviewed each article independently, and discrepancies between the two reviewers were resolved through discussion and consensus. The authors, publication years, country of investigators, sample size, total numbers for survival and death, the different detailed chemotherapy regimens, follow-up period, curative effects, adverse events, surgery plans, and the peritoneal recurrence status of each trial were extracted (Table 1). The quality of the trials was evaluated using Jadad quality scores [13], and included secure methods for randomization, allocation concealment, patient and observer blinding, and loss to follow-up. The studies were divided into a low quality group (score < 4) and high quality group (score ≥ 4) (Table 2).

**Table 1:** Basic characteristics of trials included in the present study

	**Author**	**Publication years**	**Country**	**Chemotherapy regimens**	**Chemotherapy group (number of death/total)**	**Surgery group (number of death/total)**	**Follow-uptime (months)**	**Chemotherapy group (number of R0/total)**	**Surgery group (number of R0/total)**	**Peritoneal recurrence in chemotherapy group**	**Peritoneal recurrence in surgery group**
**1**	**Koga S**[16]	1988	Japan	8-10 μg/ml MMC, 8-12 L, 50-60 min, 44-45°C	4/26	7/21	30	26/26	21/21	NA	NA
**2**	**Hamazoe R**[17]	1993	Japan	10 μg/ml MMC, 10-12 L, 50-60 min, 48-50°C	18/42	22/40	77	40/42	35/40	7*	13*
**3**	**Fujimura T**[18]	1994	Japan	30 mg MMC + 300 mg CDDP, 6-8 L,60 min, 41-42°C	7/22	14/18	36	NA	NA	2*	4*
**4**	**Ikeguchi M**[19]	1995	Japan	8-10 μg/ml MMC, 8-10 L, 50-60 min, 44-45°C	38/78	52/96	60	78/78	96/96	27*	38*
**5**	**Fujimoto S**[20]	1998	Japan	10 μg/ml MMC, 3-4 L, 120 min, 44.5-45°C	27/71	36/70	96	67/71	65/70	1*	16*
**6**	**Yonemura Y**[21]	2001	Japan	30 mgMMC + 300 mg CDDP, 6-8 L, 60 min, 42-43°C	19/48	27/47	60	48/48	47/47	6	7
**7**	**Zuo Y**[22]	2004	China	80-100 mg CDDP + 1000 mg 5-FU + 5 mg, 2 L,60 min, 41–43°C	8/46	14/36	36	NA	NA	NA	NA
**8**	**Wei G**[23]	2005	China	1000 μg/ml 5-UF, 4-5 L, 60 min, 43-45°C	21/42	25/46	36	40/49	49/55	NA	NA
**9**	**Zhang GY**[24]	2007	China	30 mg MMC + 300 mg CDDP, 2 L, 30 min, 42-45°C	44/92	75/120	60	92/92	120/120	13	45
**10**	**Deng HJ**[25]	2009	China	300-500 μg/ml 5-FU, 3 L, 60-90 min, 42-43°C	18/44	27/41	60	44/44	41/41	NA	NA

*: The number of patients dead from peritoneal recurrence.

**Table 2:** Quality assessment of trials included in the present study

	**Author**	**Randomization**	**Blind**	**Allocation concealment**	**Withdrawal and dropout**	**Jadad Scroe**
**1**	**Koga S**[16]	without details	no	well reported	well reported	4
**2**	**Hamazoe R**[17]	without details	no	well reported	well reported	4
**3**	**Fujimura T**[18]	without details	no	without details	well reported	3
**4**	**Ikeguchi M**[19]	without details	no	unclear	well reported	2
**5**	**Fujimoto S**[20]	without details	no	unclear	well reported	2
**6**	**Yonemura Y**[21]	without details	no	without details	well reported	3
**7**	**Zuo Y**[22]	without details	no	unclear	well reported	2
**8**	**Wei G**[23]	well reported	no	well reported	well reported	5
**9**	**Zhang GY**[24]	well reported	no	well reported	well reported	5
**10**	**Deng HJ**[25]	well reported	no	well reported	well reported	5

### Statistical analysis

The end-point of the meta-analysis was overall survival, defined as the time from treatment to the last follow-up or death. Results regarding the overall survival in the meta-analysis were reported as risk ratio (RR) with 95% confidence interval (CI). The heterogeneity between the trials and groups was studied using the *χ*^2^ test (or Cochran *Q* statistic) for statistical significance, and measured with *I*^2^ statistic for degree of heterogeneity [14,15]. The *I*^2^ statistic is derived from the *Q* statistic (*Q-df*/*Q* × 100). *I*^2^ < 25 was considered to indicate low heterogeneity and *I*^2^ >50% indicated a large degree of heterogeneity. If there was major heterogeneity, a random-effect model was used, and if there was no conspicuous heterogeneity, we chose a fixed-effect model for meta-analysis. The P value threshold for statistical significance was set at 0.05 for effect sizes. Publication bias was tested using the funnel plot. All statistical analysis was performed by RevMan 5.1 software, which was provided by Cochrane Collaboration.

## Results

### Eligible trials

**Figure 1:**
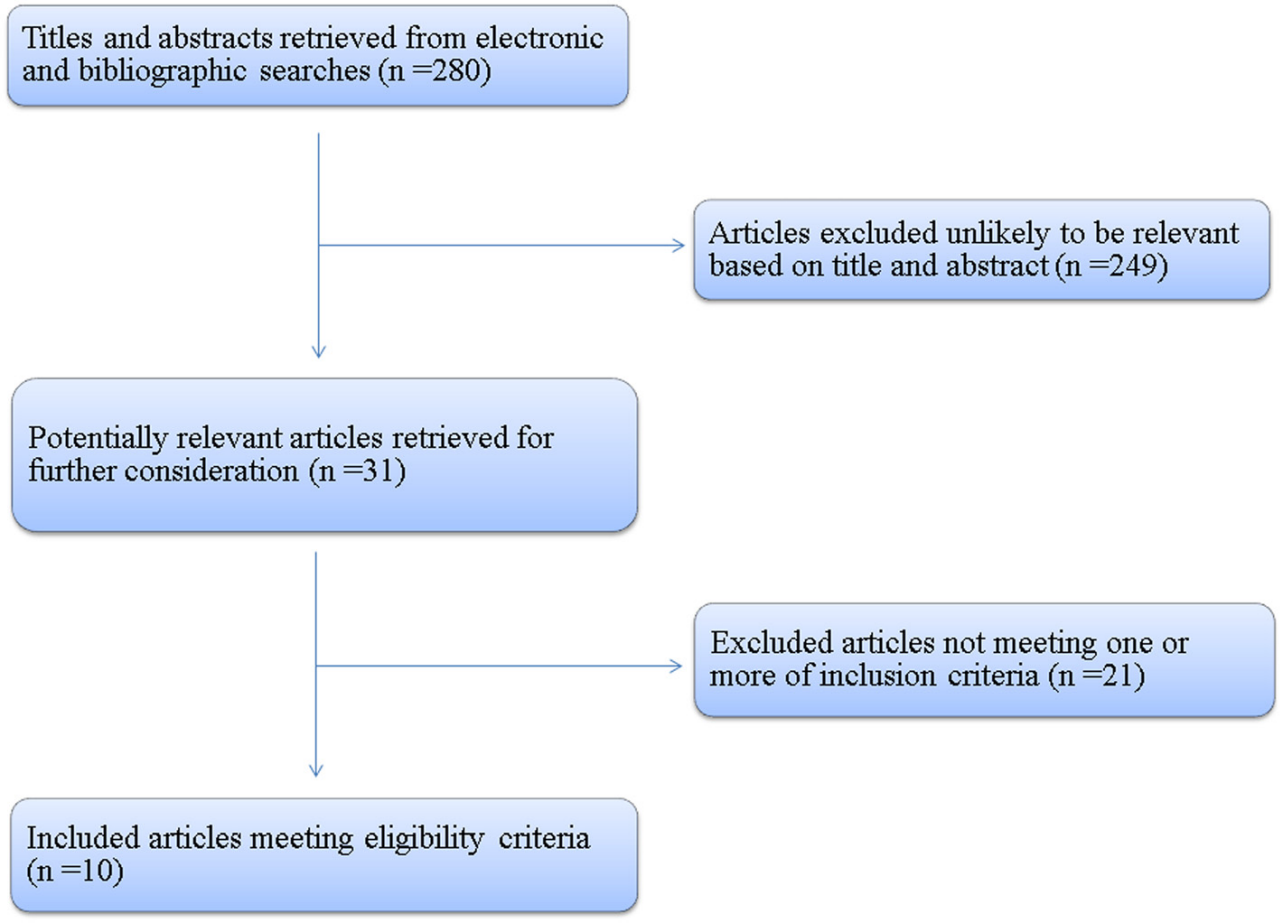
Selection of included trials.

We searched a total of 280 studies. Through screening of the titles and reading the abstracts, 31 potentially relevant reports were identified that included surgery plus HIPC versus surgery alone. Of these 31 articles, only ten randomized trials were fit the selection criteria [16-25] and included in our study. The selection procedure was further summarized in Figure 1. The 1062 gastric cancer patients enrolled in the studies were divided into the HIPC group (n = 518) and control group (n = 544), shown in Table 1. Of the ten trials, all of the investigators were from Asia: six were from Japan [16-21] and four were from China [22-25]. The quality of the included trials was evaluated according to the Jadad-scale (Table 2), and three trails [20,21,23] were low quality according to the scores (< 4 scores).

### Overall survival rates

**Figure 2:**
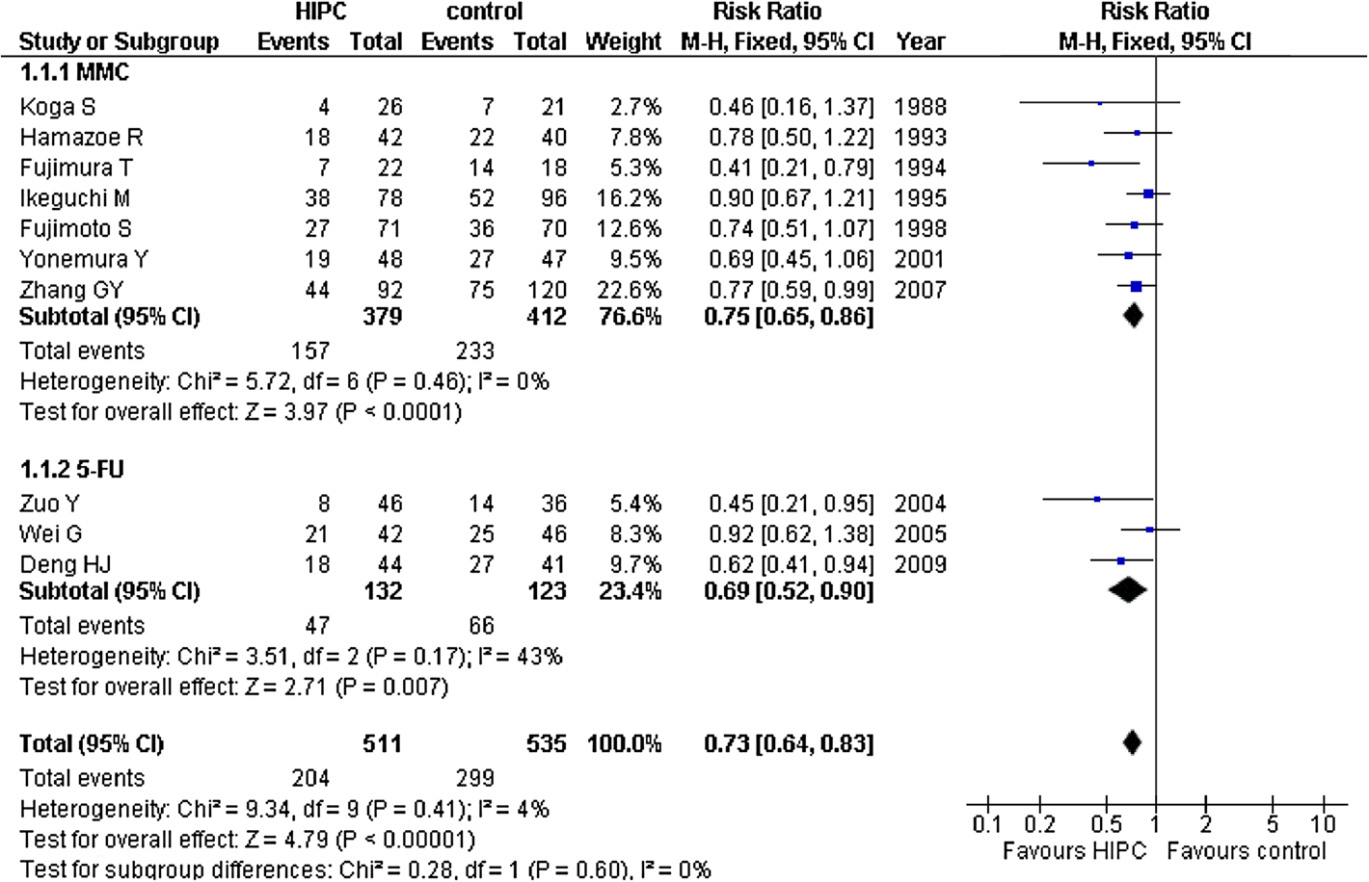
Risk Ratios for overall survival rates of all 10 randomized controlled trials.

The overall survival rate of the 1062 patients in the ten studies was shown in Figure 2[16-25] (518 in the HIPC group and 544 in the control group). Seven trials used MMC as the primary drug in HIPC [15-21] and three used 5-FU.

**Figure 3:**
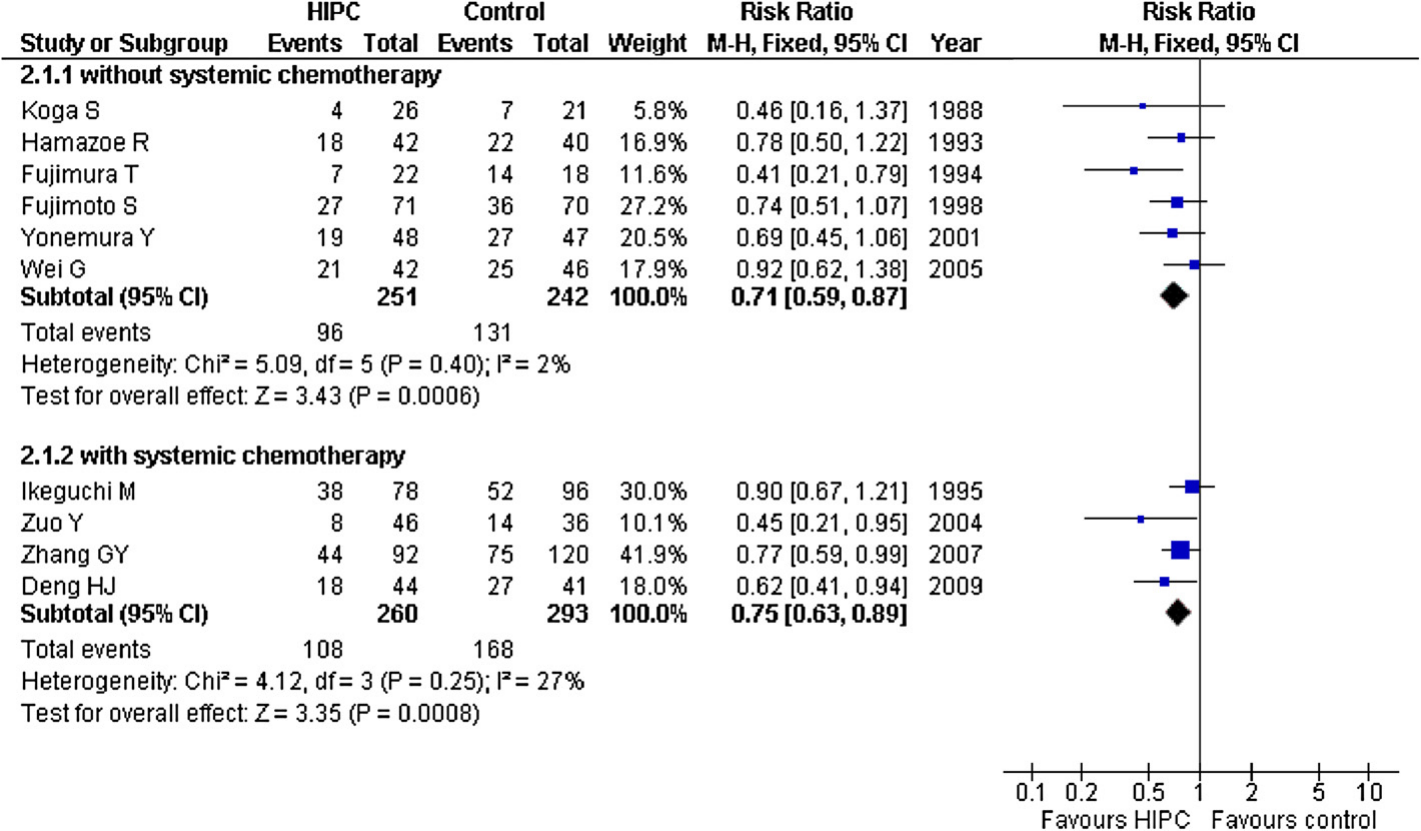
Risk Ratios for overall survival rate of trials with or without systemic chemotherapy.

Thus, we created two subgroups for analysis: MMC subgroup and 5-FU subgroup. As a result, significant survival improvements were found in the HIPC group compared to the control group, as well as in the MMC subgroup (RR = 0.75, 95%CI 0.65-0.86; P < 0.00001; fixed-effect model), and in the 5-FU group (RR = 0. 69, 95%CI 0.52-0.90; P < 0.00001; fixed-effect model). There was no obvious statistical heterogeneity in the trials. All trials analysis provided similar results (RR = 0. 73, 95%CI 0.64-0.83; P < 0.00001; fixed-effect model) without statistical heterogeneity (*I*^2^ = 0%). Four trials [19,22,24,25] utilized systemic chemotherapy after surgery for both the HIPC and control groups. We used additional analysis to obtain results that were identical in the group without systemic chemotherapy (RR = 0.71, 95%CI 0.59-0.87; P < 0.00001; fixed-effect model) and the group with systemic chemotherapy (RR = 0.75, 95%CI 0.63-0.89; P < 0.00001; fixed-effect model) (Figure 3). Sensitivity analysis was performed without the low quality trials and the results were the same (RR = 0.74, 95%CI 0.64-0.86; P < 0.00001).

### Peritoneal dissemination

There were six studies [17-21,24] that reported recurrence in the abdominal cavity (Table 1). All of these six trials described the occurrence of peritoneal local-regional recurrence, but four of them [17-20] only supplied the number of patients who died from peritoneal recurrence. Hamazoe R [17], Fujimura T [18], Ikeguchi M [19] and Fujimoto S [20] all supplied the recurrence numbers of patients who died. In Hamazoe R’s trial, 7 of 18 patients in the HIPC group died due to peritoneum local-regional recurrence, and 13 of 22 patients died in the control group. In Fujimura T’s trial, 2 of 7 patients in the HIPC group died due to peritoneum local-regional recurrence, and 4 of 14 patients died in the control group. In Ikeguchi M’s trial, 27 of 38 patients in HIPC group died due to peritoneum local-regional recurrence, and 38 of 52 patients died in the control group. Also, in Fujimoto S’s trail, 1 of 27 patients in the HIPC group died due to local recurrence, and 16 of 36 patients died in the control group. We explored the relationships among these four trials, and attempted to perform meta-analysis. However, we found that there was significant heterogeneity (P = 0.02, *I*^*2*^ = 62%); thus, we described the above four trials.

**Figure 4:**
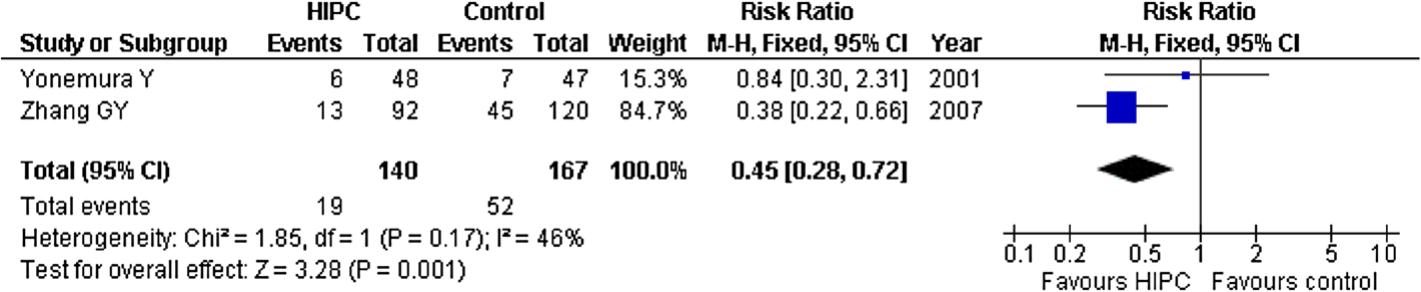
Risk Ratios for peritoneal dissemination.

The remaining two trials reported the recurrence of all patients, and we used these data for analysis. Figure 4 showed that HIPC exhibited a lower recurrence rate compared to the control group (RR = 0.45, 95%CI 0.28-0.72; P = 0.001; fixed-effect model), and the heterogeneity was not very significant between these trials. Thus, HIPC may exhibit a significant preventive effect on patients who received surgery for advanced gastric cancer.

### Adverse events

**Figure 5:**
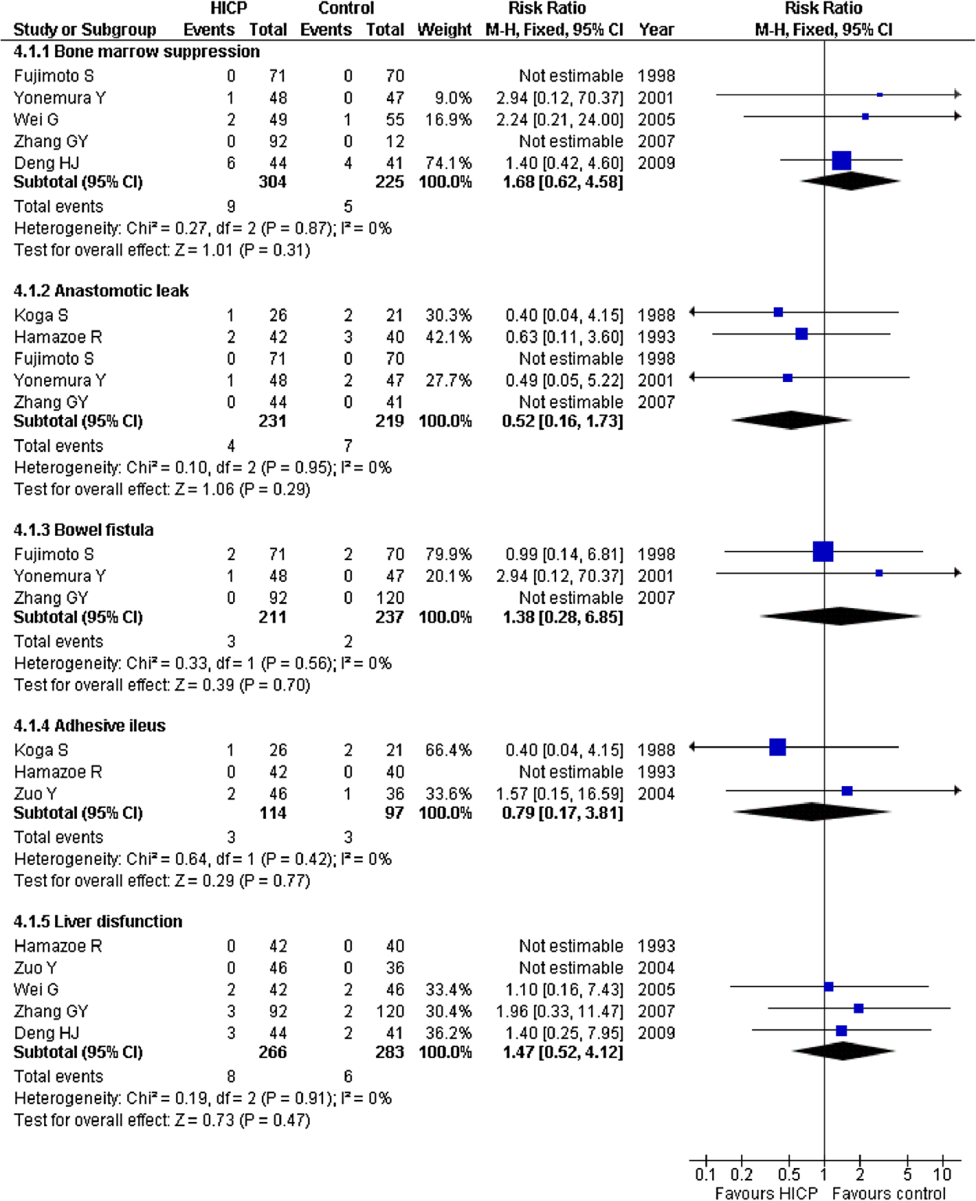
Risk Ratios for adverse events.

The adverse events included bone marrow suppression, anastomotic leak, bowel fistula, adhesive ileus, and liver disfunction. In our study, five trials [20,21,23-25] reported bone marrow suppression: one patient in the HIPC group and none in the control group in Yonemura Y’s trial, two patients in HIPC group and one in the control group in Wei G’s group, and six patients in HIPC group and four patients in the control group in Deng HJ’s study; in the two remaining trials there were no patients in either the HIPC group or control group who exhibited bone marrow suppression. The RR value was 1.68 (95%CI 0.62-4.58; P = 0.31; fixed-effect model) and there were no statistically significant differences between the HIPC and control groups. Five studies [16,17,20,21,24] reported anastomotic leak: in Koga S’s group there was one patient in the HIPC group and two patients in the control group, in Hamazoe R’s group there were two patients in the HIPC group and three patients in the control group, and in Ynoemura Y’s trial there was one patient in the HIPC group and two patients in the control group. The RR was 0.52 (95%CI 0.16-1.73; P = 0.29; fixed-effect model) and had no statistical significance. Three trials [20,21,24] reported the occurrence of bowel fistula: there were two patients in each group of Fujimoto S’s trial, one patient in the HIPC group and no patients in the control group in Ynemura Y’s report. The RR was 1.38 (95%CI 0.28-6.85; P = 0.70; fixed-effect model) and had no statistical significance. Three trials [16,17,22] recorded adhesive ileus, in which Koga S reported one patient in the HIPC group and two patients in the control group, Zuo Y described two patients in the HIPC group and one patient in the control group. The RR was 0.79 (95%CI 0.17-4.12; P = 0.77; fixed-effect model) and there was no statistical significance. Similarly, five studies reported the occurrence of liver dysfunction: in Wei G’s trial there were two patients in each group, there were three patients in the HIPC group and two patients in the control group in both trials reported by Zhang GY and Deng HJ, and the RR was 1.47 (95%CI 0.52-4.12; P = 0.47; fixed-effect model). In the other trials, there were no reports regarding the adverse events, or there were statistics of adverse events but no patients specifically cited (Figure 5). The adverse event results we obtained all exhibited no statistical significance. Because of the different research aims of the trials, the different adverse events were chosen. In the future, more comprehensive evidence is warranted.

## Discussion

The survival rate of patients with gastric cancer has improved along with the improvement of surgical procedures [26]. However, many patients who have received gastric cancer resection still have suffered local-regional or peritoneal recurrence. Dissemination of free tumor cells through blood or lymph into the abdominal cavity has been considered one of the most common causes of peritoneal dissemination of gastric cancer [27]. Thus, it is very important to eliminate the free tumor cells in the abdominal cavity in order to improve survival rates. Intraperitoneal chemotherapy for intraperitoneal cancer was first suggested in the 1950s [28]. Intraperitoneal chemotherapy is able to kill the free tumor cells left behind after surgery that were not eliminated by traditional systemic chemotherapy. Hyperthermic intraperitoneal chemotherapy was first performed as a clinical trial to investigate removal of intraperitoneal tumor cells in 1980 [12]. HIPC washes out intraperitoneal free tumor cells using a large massive liquid, and damages cancer cells or micrometastases directly due to the heat sensitivity of tumor cells [17,29]. Thus, HIPC combined with surgery has been used to control peritoneal metastasis in gastric cancer; however, there is still no solution as to whether it exhibits an effect on long-term survival and prevention of peritoneal recurrence.

**Figure 6:**
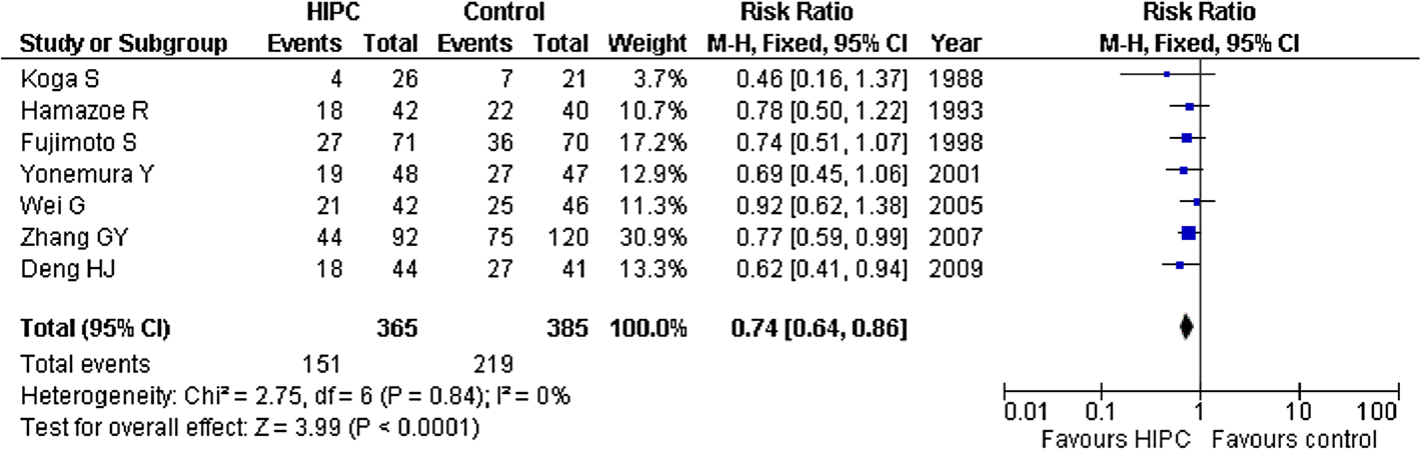
Sensitivity analysis for overall survival: high-quality studies (Jadad score ≥≥≥ 4).

**Figure 7:**
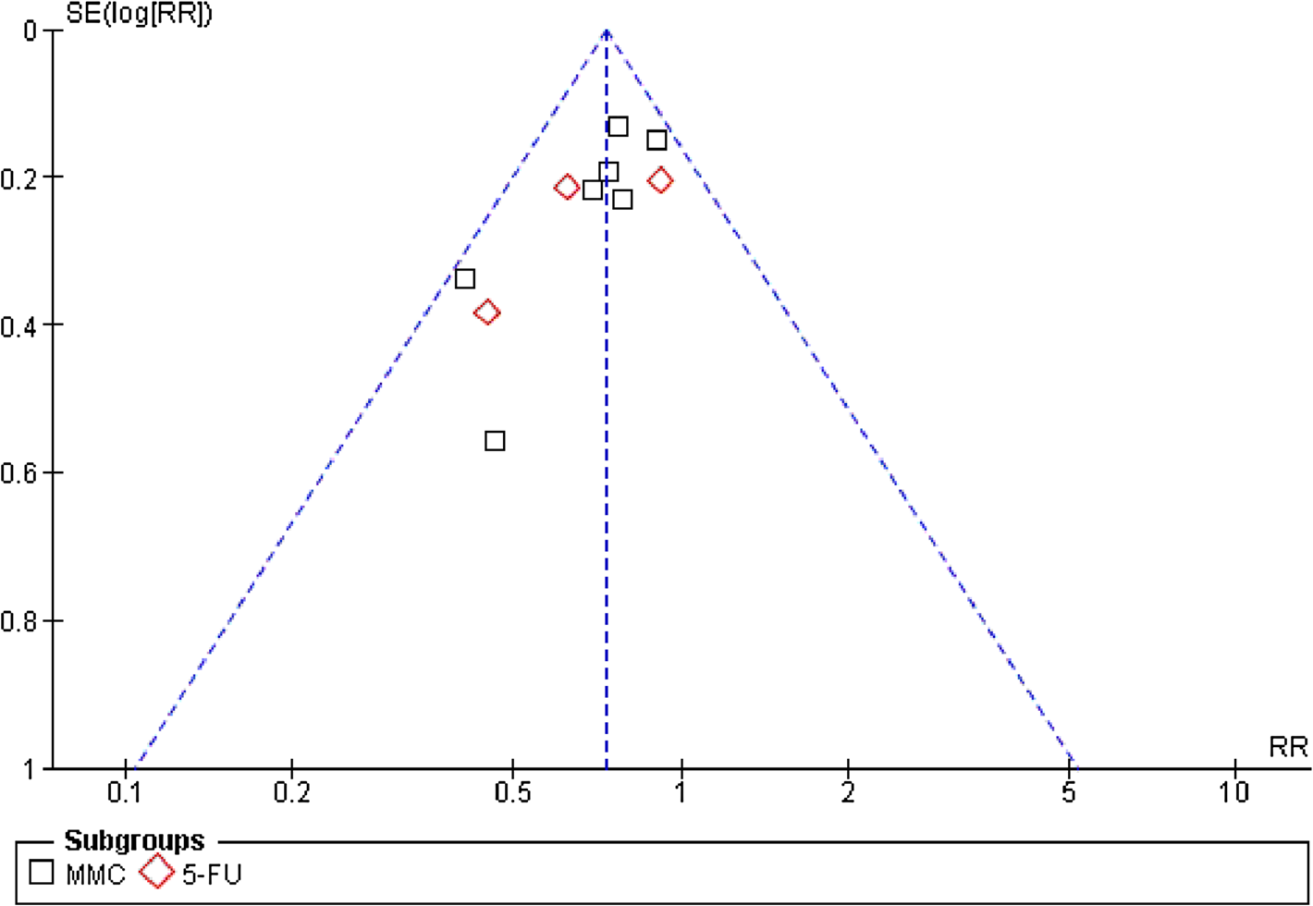
Funnel plot analysis of potential publication bias.

The purpose of a meta-analysis is to supply an exhaustive and neoteric summary of all relevant RCTs concerning the topic, and to provide guidance for future clinical work. Our present meta-analysis demonstrated the effects of HIPC in correlation with different chemotherapy regimens for patients who had received advanced gastric cancer tumor resection in order to improve the survival rate. In a study conducted by Xu DZ et al. [10], intraperitoneal chemotherapy after cancer was demonstrated to be beneficial to patients with gastric cancer. Similar results were reported with six RCTs on the topic by Yan TD et al. [8] in 2007, in which it was demonstrated that hyperthermic intraoperative intraperitoneal chemotherapy (HIIC) with or without early postoperative intraperitoneal chemotherapy (EPIC) following resection of gastric cancer improved survival. Sensitivity analyses (Figure 6) and funnel plot analyses concerning potential publication bias (Figure 7) were also performed to confirm the reliability of our research results. The publication bias may be a problem for meta-analysis, but we did not find this bias in our study.

In the past, many researchers have reported that approximately 50% of patients who received resection of advanced gastric cancer exhibited a local-regional recurrence in their abdominal cavity and a poor prognosis [30,31]. However, because few RCTs reported peritoneal recurrence, the earlier studies could not be utilized to analyze the relationship between the HIPC and control groups regarding local-regional recurrence. We included six trials that reported peritoneal local-regional recurrence, but only two trials supplied the recurrence rate for all patients. The results of our meta-analysis indicated that HIPC could potentially allow for a better prognosis in patients who underwent resection for advanced gastric cancer compared to the control group, and may play a role in the prevention of peritoneal local-regional recurrence. The other four trials, except for the report by Ikeguchi M [19], all indicated that peritoneal recurrence was more frequent in the control group compared to the HIPC group. Additionally, Ikeguchi M reported that HIPC potentially prevents peritoneal metastases among patients with no lymph node metastases. We also counted the type of radical surgery received by the patients (Table 1), and a great majority of patients received R0 resection. It was interesting that patients of the two trails processed in meta-analysis were all received R0 radical resection. Thus, for gastric cancer patients (serosal invasion) with R0 resection but high probability of peritoneal recurrence, HIPC may play an important role. However, the small number of trials that have reported total peritoneal recurrence is a problem that we encountered; the study of a greater number of interrelated studies is awaited.

The adverse events in the HIPC and control groups were also described in our research. The independent effects of hyperthermia for cancer cells is strongly increased when the temperature range is 42.5°C to 43.0°C, but because selective heating of only tumor cells is very difficult the injury to normal tissues is also increased [18]. In Yan TD’s [8] study, he reported that HIIC with or without EPIC after resection of gastric cancer increased the risk of intra-abdominal abscess and neutropenia. Few clinical trials have previously described the side effects of HIPC, and in various reports differing data were supplied. Our results indicated that there were no statistically significant differences regarding adverse events between the HIPC group and the control group. However, further comprehensive proof is needed to confirm this.

Over the past couple of decades, several investigators reported that HIPC significantly improved the survival rate in serosa-invasive gastric cancer patients. This finding was due to the prevention of early postoperative peritoneal metastasis [26]. However, although our findings indicated that the survival rate might be generally significantly improved following HIPC, the individual optimal regimen remains unclear, and further studies are warranted. Also, the effectiveness of HIPC potentially depends on the diameter and depth of the micrometastasis, because heat or drugs cannot reach the cancer cells [16,32,33]. However, there were several adverse events after the treatment of HIPC that were not avoided as common complications of chemotherapy. In spite of the Jadad-scale was used to assess the investigations and all articles included in the studies were RCT, the quality of RCT studies cannot be fully accessed. The bias caused by quality of included articles may be a factor which may influence the result of the study. Although the Jadad-scale is visualized and pellucid, a consummate and exhaustive appraisal procedure is still awaited. Additionally, all of the trials included in this analysis were from Asia, particularly China and Japan. Thus, whether HIPC is useful for other patients of the world remains to be seen in further investigations.

## Conclusion

In conclusion, our meta-analysis demonstrated that HIPC potentially improves the overall survival rate of patients who underwent resection for advanced gastric cancer, and potentially functions by preventing local recurrence. In the future, higher quality studies, superior patient selection, and well designed multi-center RCTs are awaited.

## Abbreviations

5-FU: 5-fluorouracil; CCTR: Cochrane Central Register of Controlled Trials; CDDP: Cisplatin; CDSR: Cochrane Database of Systematic Reviews; CI: Confidence Interval; EPIC: Early Postoperative Intraperitoneal chemotherapy; HIIC: Hyperthermic Intraoperative Intraperitoneal Chemotherapy; HIPC: Hyperthermic Intraperitoneal Chemotherapy; IPC: Intraperitoneal Chemotherapy; MMC: Mitomycin C; RR: Relative Risk; GC: Gastric Cancer.

## Competing interests

The authors declare that they have no competing interests.

## Authors’ contributions

JS and YS contributed equally to this work. ZW participated in the conception and design of the study and coordination; JS and YS participated in design of the study, data extraction, article selection and manuscript preparation and interpreted the results in collaboration with YX and HX; JL and XC participated in data extraction, article selection and data extraction; PG performed the statistical analysis and participated in the critical revision of the manuscript. All authors drafted and critically revised the manuscript and approved the final version.

## Pre-publication history

The pre-publication history for this paper can be accessed here:

http://www.biomedcentral.com/1471-2407/12/526/prepub

## References

[B1] Bertuccio P Chatenoud L Levi F Praud D Ferlay J Neqri E Malvezzi M La VC Recent patterns in gastric cancer: A global overview Int J Cancer 2009 125 666 673 10.1002/ijc.24290 19382179

[B2] Parkin DM Bray F Ferlay J Pisani P Global cancer statistics, 2002 CA Cancer J Clin 2005 55 2 74 108 10.3322/canjclin.55.2.74 15761078

[B3] Crew KD Neugut AI Epidemiology of gastric cancer World J Gastroenterol 2006 12 3 354 362 16489633 10.3748/wjg.v12.i3.354 PMC4066052

[B4] Hartgrink HH Jansen EP van Grieken NC van de Velde CJ Gastric cancer Lancet 2009 374 9688 477 490 10.1016/S0140-6736(09)60617-6 19625077 PMC4613761

[B5] Sadeghi B Arvieux C Glehen O Beaujard AC Rivoire M Baulieux J Fontaumard E Brachet A Caillot JL Faure JL Peritoneal carcinomatosis from non-gynecologic malignancies: Results of the EVOCAPE 1 multicentric prospective study Cancer 2000 88 358 363 10.1002/(SICI)1097-0142(20000115)88:2<358::AID-CNCR16>3.0.CO;2-O 10640968

[B6] Roviello F Marrelli D Manzoni GD Morqaqni P Di Leo A Saraqoni L De Stefano A Prospective study of peritoneal recurrence after curative surgery for gastric cancer Br J Surg 2003 90 1113 1119 10.1002/bjs.4164 12945079

[B7] Yamada E Miyaishi S Nakazato H The surgical treatment of cancer of the stomach Int Surg 1980 65 387 399 6161095

[B8] Yan TD Black D Suqarbaker PH Zhu J Yonemura Y Petrou G Morris DL A systematic review and meta-analysis of the randomized controlled trials on adjuvant intra-peritoneal chemotherapy for resectable gastric cancer Ann Surg Oncol 2007 14 2702 2713 10.1245/s10434-007-9487-4 17653801

[B9] Sugarbaker PH Yu W Yonemura Y Gastrectomy, peritonectomy, and perioperative intraperitoneal chemotherapy: The evolution of treatment strategies for advanced gastric cancer Semin Surg Oncol 2003 21 233 248 10.1002/ssu.10042 14648781

[B10] Xu DZ Zhan YQ Sun XW Cao SM Geng QR Meta-analysis of intraperitoneal chemotherapy for gastric cancer World J Gastroenterol 2004 10 18 2727 2730 15309728 10.3748/wjg.v10.i18.2727 PMC4572202

[B11] Shiu MH Fortner JG Intraperitoneal hyper-thermic treatment of implanted peritoneal can-cer in rats Cancer Res 1980 40 4081 4084 7471053

[B12] Spratt JS Adcock RA Muskovin M Sherrill W McKeown J Clinical delivery system for intraperitoneal hyperthermic chemotherapy Cancer Res 1980 40 256 260 6766084

[B13] Jadad AR Moore RA Carroll D Jenkinson C Reynolds DJ Gavaghan DJ McQuay HJ Assessing the quality of reports of randomized clinical trials: is blinding necessary? Control Clin Trials 1996 17 1 12 10.1016/0197-2456(95)00134-4 8721797

[B14] Cochran WG The combination of estimates from different experiments Biometrics 1954 10 101 129 10.2307/3001666

[B15] Higgins JPT Thompson SG Deeks JJ Altman DG Measuring inconsistency in meta-analyses BMJ 2003 327 7414 557 560 10.1136/bmj.327.7414.557 12958120 PMC192859

[B16] Koga S Hamazoe R Maeta M Shimizu N Murakami A Wakatsuki T Prophylactic cancer therapy for peritoneal recurrence of gastric by continuous hyperthermic peritoneal perfusion with Mitomycin C Cancer 1988 61 2 232 237 10.1002/1097-0142(19880115)61:2<232::AID-CNCR2820610205>3.0.CO;2-U 3121165

[B17] Hamatoe R Maeta M Kaibara N lntraperitoneal thermochemotherapy for prevention of peritoneal recurrence of gastric cancer Cancer 1994 73 8 2048 2052 10.1002/1097-0142(19940415)73:8<2048::AID-CNCR2820730806>3.0.CO;2-Q 8156509

[B18] Fujimura T Yonemura Y Muraoka K Continuous hyperthermic peritoneal perfusion for the prevention of peritoneal recurrence of gastric cancer: randomized controlled study Wrold J Surg 1994 18 1 150 155 10.1007/BF00348209 8197772

[B19] Ikeguch M Kondou A Oka A Effects of continuous hyperthermic peritoneal perfusion on prognosis of gastric cancer with serosal invasion Eur J Surg 1995 161 8 581 586 8519874

[B20] Fujimoto S Takahashi M Mutou T Kobayashi K Toyosawa T Successful intra-peritoneal hyperthermic chemoperfusion for the prevention of postoperative peritoneal recurrence in patients with advanced gastric carcinoma Cancer 1999 85 529 554 10.1002/(SICI)1097-0142(19990201)85:3<529::AID-CNCR3>3.0.CO;2-9 10091726

[B21] Yonemura Y de Aretxabala X Fujimura T Intraoperative chemohyperthermic peritoneal perfusion as an adjuvant to gastric cancer: final results of a randomised controlled study Hepatogastroenterol 2001 48 1776 1782 11813623

[B22] Zuo Y Xu M Shen D Lu JF Postoperative intraperitionealhyperthermic chemoperfusion combined with intravenous chemotherapy for 82 advanced gastric cancer patients Zhonghua Zhongliu Zazhi 2004 26 247 249 15312391

[B23] Wei G Fang GE Bi JW Shen XJ Nie MM Xue XC Hua JD Efficacy of intraoperative hypotonic peritoneal chemo-hyperthermia combined with early postoperative intraperitoneal chemotherapy on gastric cancer Ai Zheng 2005 24 478 482 15820074

[B24] Zhang GY Chen XC Pan K Xia LG Zuo M Zheng T Application of hyperthermic intraoperitoneal chemotherapy in patients with gastric cancer Zhonghua Wei Chang Waike Zazhi 2007 10 4 362 364 17659464

[B25] Deng HJ Wei ZG Zhen L Li GX Uang XC Qing SH Clinical application of perioperative continuous hyperthermic peritoneal perfusion chemotherapy for gastric cancer Nan Fang Yi Ke Da Xue Xue Bao 2009 29 2 295 297 19246304

[B26] Kim JY Bae HS A controlled clinical study of serosa-invasive gastric carcinoma patients who underwent surgery plus intraperitoneal hyperthermo-chemo-perfusion (IHCP) Gastric Cancer 2001 4 27 33 10.1007/s101200100013 11706624

[B27] Sugarbaker PH Management of Gastric Cancer 1991 Boston: Kluwer Academic Publisher 277 284

[B28] Weisberger AS Levine B Storaasli JP Use of nitrogen mus-tard in treatment of serous effusions of neoplastic origin JAMA 1955 159 1704 1707 10.1001/jama.1955.02960350004002 13271133

[B29] Crile G Jr: Selective destruction of cancers after exposure to heat Ann Surg 1962 156 404 407 10.1097/00000658-196209000-00007 13882218 PMC1466188

[B30] MacDonald JS Malley SR Benedetti J Hundahl SA Estes NC Stemmermann GN Haller DG Ajani JA Gunderson LL Jessup JM Chemoradiotherapy after surgery compared with surgery alone for adenocarcinoma of the stomach or gastroesophageal junction N Engl J Med 2001 345 725 729 10.1056/NEJMoa010187 11547741

[B31] Cunningham D Allum WH Stenning SP Thompson JN Van de Velde CJ Nicolson M Scrarffe JH Lofts FJ Falk SJ Iveson TJ Perioperative chemotherapy versus surgery alone for resectable gastro-esophageal cancer N Engl J Med 2006 355 11 20 10.1056/NEJMoa055531 16822992

[B32] Kazuo H Kanji K Atsushi I Yamaquchi A Nakaqawara G Umeda S Kusaka Y Efficacy of Continuous Hyperthermic Peritoneal Perfusion for the Prophylaxis and Treatment of Peritoneal Metastasis of Advanced Gastric Cancer: Evaluation by Multivariate Regression Analysis Oncology 1999 57 106 114 10.1159/000012016 10461056

[B33] Sugarbaker PH Intraperitoneal chemotherapy and cytoreductive surgery for the prevention and treatment of peritoneal carcinomatosis and sarcomatosis Semin Surg Oncol 1998 14 3 254 261 10.1002/(SICI)1098-2388(199804/05)14:3<254::AID-SSU10>3.0.CO;2-U 9548609

